# Pericyte detachment and renal congestion involve interstitial injury and fibrosis in Dahl salt-sensitive rats and humans with heart failure

**DOI:** 10.1038/s41440-023-01451-3

**Published:** 2023-10-16

**Authors:** Hiroki Ito, Takuo Hirose, Shigemitsu Sato, Chika Takahashi, Risa Ishikawa, Akari Endo, Ayaka Kamada, Ikuko Oba-Yabana, Tomoyoshi Kimura, Kazuhiro Murakami, Yasuhiro Nakamura, Kazuhiro Takahashi, Takefumi Mori

**Affiliations:** 1 Division of Nephrology and Endocrinology, Faculty of Medicine, Tohoku Medical and Pharmaceutical University, Sendai, Japan; 2 Department of Endocrinology and Applied Medical Science, Tohoku University Graduate School of Medicine, Sendai, Japan; 3 Division of Integrative Renal Replacement Therapy, Faculty of Medicine, Tohoku Medical and Pharmaceutical University, Sendai, Japan; 4 Division of Pathology, Tohoku Medical and Pharmaceutical University, Sendai, Japan

**Keywords:** Dahl salt-sensitive rats, Venous congestion, Renal congestion, Pericyte detachment, Cardiorenal syndrome

## Abstract

Congestive heart failure produces fluid volume overload, central and renal venous pressure elevation, and consequently renal congestion, which results in worsening renal function. Pericyte detachment and pericyte-myofibroblast transition (PMT) were linked to renal interstitial fibrosis. Dahl salt-sensitive hypertensive (DahlS) rats are a non-surgical renal congestion model. The relation, however, between renal interstitial damage, pericyte morphology, and PMT in the renal congestion of DahlS rats has not been reported. DahlS rats (8-week-old) were fed normal salt (NS, 0.4% NaCl) or high salt (HS, 4% NaCl), and the left kidney was decapsulated to reduce renal interstitial hydrostatic pressure (RIHP) at 9 weeks old. One week after capsulotomy, both kidneys were analyzed by molecular and histological techniques. Renal pericyte structure was assessed in the body donors with/without venous stasis. Markers of tubulointerstitial damage, interstitial fibrosis, and PMT were upregulated in the right non-decapsulated kidney of DahlS rats fed HS. Renal tubular injury and fibrosis were detected in the HS diet groups in histological analysis. Pericyte detachment was observed in the right non-decapsulated kidney of DahlS rats fed HS by low vacuum-scanning electron microscopy. Decapsulation in DahlS rats fed HS attenuated these findings. Also, renal pericytes detached from the vascular wall in patients with heart failure. These results suggest that pericyte detachment and PMT induced by increased RIHP are responsible for tubulointerstitial injury and fibrosis in DahlS rats and humans with renal congestion. Renal venous congestion and subsequent physiological changes could be therapeutic targets for renal damage in cardiorenal syndrome.

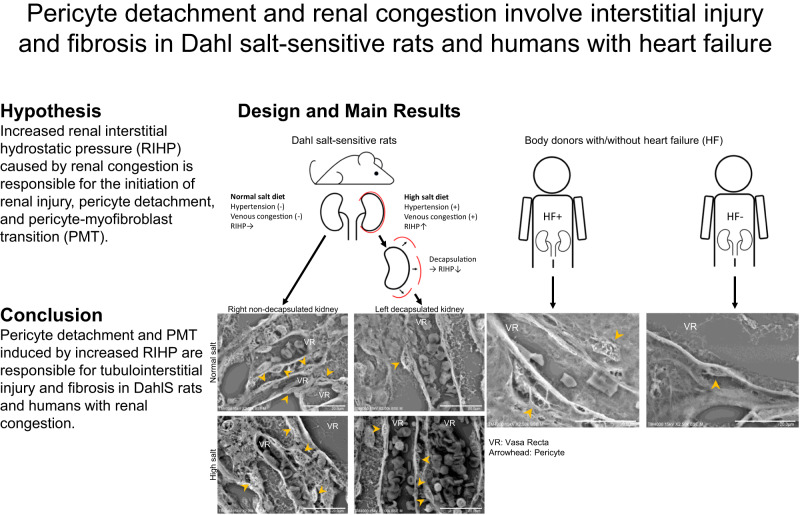

## Introduction

Management of heart failure complications is of great interest, because the incidence of heart failure is expected to increase at pandemic levels, especially in the elderly [1]. One of the severe complications of heart failure is the hemodynamic impact on renal function, which is called cardiorenal syndrome [2]. Congestive heart failure produces a state of fluid volume overload, increased right atrial pressure, central venous pressure (CVP) elevation, and consequently renal venous pressure (RVP) elevation [3]. Renal venous congestion due to abdominal venous stasis is closely associated with cardiorenal syndrome and relates to the progression of heart failure [4–6]. CVP is correlated with renal function in patients with chronic heart failure and is a strong predictor of mortality [7]. The elevation of RVP raises renal interstitial hydrostatic pressure (RIHP), causes compression of renal peritubular capillaries, and decreases renal blood flow [8, 9]. Moreover, the elevation of RVP is one of the causes of renal fibrosis via epithelial-mesenchymal transition [10, 11].

We have developed a novel rat unilateral renal venous congestion model, in which RVP of the left kidney was artificially elevated by ligating the inferior vena cava (IVC) between both renal veins [12, 13]. In this model, RHIP elevation, tubular dilatation, and interstitial fibrosis were observed in the left congested kidney. In addition, the luminal descending vasa recta was expanded, but the peritubular capillaries were less expanded in the congested kidney. Pericyte detachment was also present, which was accompanied by excessive accumulation of collagen and other extracellular matrix components. Pericyte detachment from capillaries following acute or chronic kidney damage would be crucial in the progression of chronic kidney disease [14, 15] and interstitial fibrosis through pericyte-myofibroblast transition (PMT) [16–20]. Indeed, PMT and tubular injury markers were upregulated in the left congested kidney [12]. And this renal congestion-derived tubulointerstitial injury was ameliorated by RIHP reduction through renal capsulotomy [12] or inhibition of the platelet-derived growth factor receptor (PDGFR) pathway [13], which causes PMT upon activation [21].

Dahl salt-sensitive hypertensive (DahlS) rats on high salt (HS) diet exhibit hypertension, congestive heart failure, fluid volume overload, and RVP elevation, which are considered to be a non-artificial and -surgical renal congestion model [22–26]. DahlS rats had renal injury and fibrosis due to renal congestion [11, 27, 28]. The relation, however, between renal interstitial injury and fibrosis, pericyte detachment, and PMT in DahlS rats has not been reported.

We, therefore, hypothesized that increased RIHP caused by renal congestion is responsible for the initiation of renal injury, pericyte detachment, and PMT in DahlS rats. To test this hypothesis, we investigated the relation of pericyte detachment from the vasa recta and RIHP reduction by renal decapsulation with interstitial injury and fibrosis in DahlS rats. In addition, due to the lack of evidence for pericyte loss or detachment in the human kidneys, we examined the pericyte structure in the kidneys of patients with and without heart failure.

## Methods

### Animals

All animal experiments conducted in this study were in accordance with the National Institutes of Health Guide for the Care and Use of Laboratory Animals, and were approved by the Tohoku Medical and Pharmaceutical University Animal Experiment Committee (registration numbers: A18019-a, A19039-cn, A20005-cn, A21008-cn, and A22042-cn). Male DahlS rats (SS/Jr/Mcwi) were obtained from a closed colony maintained at the Center for Laboratory Animal Science of Tohoku Medical and Pharmaceutical University. Male spontaneously hypertensive rats (SHR/Izm) were purchased from Japan SLC (Shizuoka, Japan). The rats were housed in environmentally controlled rooms under a 12-hour light/dark cycle and had access to tap water *ad libitum*. Eight-week-old DahlS rats were randomly divided into two groups; normal salt (NS; 0.4% NaCl diet) and HS (4% NaCl diet) for 2 weeks as described previously [11]. SHR were kept for at least 5 days for acclimation with a standard pellet chow (CE-2; CLEA Japan, Tokyo, Japan). Animal experiments were designed to use all animals for the analysis, except for those that died or suffered from issues related to animal welfare throughout the experiment.

### Human kidney samples

The institutional ethics committee of Tohoku Medical and Pharmaceutical University Hospital reviewed and approved human tissue collection and all experimental protocols (registration number: 2021-2-109). We enrolled the kidneys of all patients whose pathological anatomy was performed in our department from September 2017 to March 2023. Written informed consent for the use of post-mortem tissue analysis was obtained from the family members of the decedent. Tissues were fixed in 10% neutralized buffered formalin and embedded in paraffin wax. Clinical data were collected from their medical records. The diameter of the IVC was measured from images taken by computed tomography. Heart failure was determined according to the cardiopathological findings at autopsy and the JCS/JHFS 2021 guidelines [29].

### Renal decapsulation

All DahlS rats fed NS or HS received the left renal capsulotomy on day 7 (Supplementary Fig. 1), as described in our previous report [12] with slight modification. Briefly, rats were placed on a temperature-controlled (38°C) operating table and anesthetized with three types of mixed anesthetic agents (0.15 mg/kg of medetomidine, Maruishi Pharmaceutical, Osaka, Japan; 2.0 mg/kg of midazolam, Astellas Pharma, Tokyo, Japan; and 2.5 mg/kg of butorphanol, Meiji Seika Pharma, Tokyo, Japan) intramuscularly. For decapsulation, the left abdomen was incised to expose the left kidney, and the left renal capsule was completely removed. The kidneys were then returned to the retroperitoneal space, and the abdominal walls and skin were closed. Blood pressure was measured by the tail-cuff method after 7 days of capsulotomy and subsequently anesthetized under the aforementioned anesthesia. After drawing blood from the abdominal aorta, the rats were euthanized to remove the kidneys and heart. SHR at 10-, 12-, and 14-week-olds were euthanized under anesthesia, and the kidneys were removed.

The removed tissues were immediately weighed and sectioned. The tissues were fixed with 10% neutralized buffered formalin (Mildform; Wako Pure Chemical Industries, Osaka, Japan) and embedded in paraffin for histological analysis. Both kidney sections were separated into the part of the cortex and outer medulla, and preserved in RNA Later (Invitrogen, Carlsbad, CA) for RNA or snap-frozen in liquid nitrogen for protein analysis. The biochemical tests were performed by Nagahama Life Science Laboratory (Nagahama, Japan).

### RNA expression level quantification

Total RNA was isolated from the kidney tissues using ISOGEN (NIPPON GENE, Tokyo, Japan) and relative mRNA levels were analyzed by reverse transcription real-time quantitative polymerase chain reaction (RT-qPCR) as previously described [13, 30]. Briefly, PrimeScript reverse transcriptase (TaKaRa Bio, Shiga, Japan) and random hexamers (Invitrogen) were used to synthesize cDNA from the total RNA. The target cDNAs were amplified in duplicate by THUNDERBIRD Next SYBR qPCR Mix (Toyobo, Osaka, Japan) and gene-specific primers (Supplementary Table 1) using CFX Connect (Bio-Rad, Hercules, CA). The relative mRNA expression levels were standardized to the values of *peptidylprolyl isomerase A* (*Ppia*), *phosphoglycerate kinase 1* (*Pgk1*), and *ribosomal protein lateral stalk subunit P2* (*Rplp2*).

### Western blot analysis

The kidney tissues were homogenized in a mixture of lysis buffer (9803; Cell Signaling Technology, Danvers, MA), 1.0 mmol/L phenylmethylsulfonyl fluoride (Thermo Fisher Scientific), and a protease inhibitor cocktail (Roche, Basel, Switzerland), as previously described [13, 30]. Twenty µg of proteins mixed with Laemmli sample buffer (Bio-Rad) and 2.5% mercaptoethanol were separated through 4-20% Mini-PROTEAN TGX Gels (Bio-Rad) and transferred onto trans-blot turbo transfer pack membranes (Bio-Rad). After blocking nonspecific bindings by PVDF Blocking Reagent for Can Get Signal (Toyobo), the membranes were incubated with antigen-specific antibodies (Supplementary Table 2) overnight at 4°C. The signals of the immunoreaction were visualized using a horseradish peroxidase-conjugated secondary antibody (1:5,000; Cell Signaling Technology), an enhanced chemiluminescence system (Clarity Western ECL Substrate; Bio-Rad), and a chemiluminescent detection system (WSE-6300H LuminoGraph III; ATTO, Tokyo, Japan). Glyceraldehyde-3-phosphate dehydrogenase (GAPDH) was used to normalize the relative expression level of each protein.

### Histological analysis

Four-µm-thick sections for rat kidneys and 1.5-µm-thick sections for human kidneys were deparaffinized with xylene and hydrated with gradient ethanol and distilled water. The sections were routinely stained with hematoxylin-eosin (HE) and Elastica-Masson (EM) for routine histological analysis in the Technical Service Division of Tohoku Medical and Pharmaceutical University.

For immunostaining, heat-induced antigen retrieval was performed in an autoclave for 5 min at 121°C in 10 mmol/L citrate buffer (pH 6.0) or 1.0 mmol/L ethylenediaminetetraacetic acid buffer (pH 9.0) after deparaffinization, as previously described [13, 30]. Then, the antigens were reacted with antigen-specific antibodies (Supplementary Table 2) overnight at 4°C. The next day, the sections were incubated with an immune-enzyme polymer (Histofine Simple Stain MAX PO; Nichirei Biosciences, Tokyo, Japan) and 3,3′-diaminobenzidine (DAB, SK-4100; Vector Laboratories, Newark, CA), or fluorophore-conjugated secondary antibodies (Alexa 488 and 555; Molecular Probes, Carlsbad, CA). The nuclei were counterstained with hematoxylin or Hoechst 33342 (Molecular Probes). The slides were digitized by a bright-field slide scanner system (NanoZoomer-SQ; Hamamatsu Photonics, Hamamatsu, Japan) or laser scanning confocal microscopy (Leica TCS SP8; Leica Microsystems, Wetzlar, Germany).

### Low-vacuum scanning electron microscopy

Low-vacuum scanning electron microscopy (LV-SEM, Miniscope TM4000; Hitachi High-Technologies, Tokyo, Japan) was used to examine the ultrastructure of the vasa recta, as previously reported [12, 13]. Briefly, 4-µm-thick sections were stained with Pt-blue solution (TI-blue small kit; Nisshin EM, Tokyo, Japan) and captured at an acceleration voltage of 15 kV and a chamber pressure of 30 Pa.

### Pericyte detachment score in human and rat kidney samples

Two well-trained renal pathologists randomly selected 4 fields from images of human and rat kidneys in LV-SEM, and scored levels of pericyte detachment on a four-point scale (- (0%), +/- (0-10%), + (10-50%), and 2+ (50-100%)).

### Statistical analysis

Data collection, interpretation, and statistical analysis were carried out by separate researchers with blinded procedures. The continuous values were presented as the mean ± standard error of the mean (SEM). Statistical comparisons were conducted using Fisher’s exact test for categorical data involving small numbers (pericyte detachment score in rats), unpaired *t*-test for two-group comparisons, and one-way analysis of variance (ANOVA) followed by Tukey’s honestly significant difference test for multiple comparisons. *P* values < 0.05 were considered statistically significant.

## Results

### Biometric, biochemical, and morphological analysis

No rats died and had animal welfare issues during the experimental period. The mean values for body and tissue weights, blood pressure, and biochemical parameters were listed in Table 1. The kidney weight/body weight ratio was significantly elevated by HS intake. This ratio was not different between the right contralateral non-decapsulated and left decapsulated kidneys in both NS and HS groups, due to new capsule formation on the left decapsulated kidney in all DahlS rats used. DahlS rats fed HS showed higher blood pressure than those fed NS. Serum creatinine (Cr) and blood urea nitrogen (BUN) showed no significant differences between the groups.

**Table 1:** Biometric and biochemical analysis

	Normal salt	High salt
*n*	8	7
sBP (mm Hg)	147.2 ± 3.2	171.3 ± 6.8*
HR (beats/min)	409.0 ± 6.2	416.0 ± 9.4
BW (mg)	268.9 ± 13.2	278.3 ± 9.1
Heart (mg/g BW)	3.72 ± 0.06	4.16 ± 0.15*
Kidney^a^		
Right (mg/g BW)	4.22 ± 0.10	5.23 ± 0.21^†^
Left (mg/g BW)	3.99 ± 0.06	5.14 ± 0.18^†^
Serum		
TP (g/dL)	5.61 ± 0.11	5.40 ± 0.18
Alb (g/dL)	3.83 ± 0.07	3.49 ± 0.12
BUN (mg/dL)	20.0 ± 0.8	21.7 ± 1.3
Cr (mg/dL)	0.34 ± 0.03	0.32 ± 0.04
UA (mg/dL)	0.69 ± 0.17	1.37 ± 0.64
Na (mEq/L)	136.5 ± 0.8	135.3 ± 0.7
K (mEq/L)	4.71 ± 0.25	5.10 ± 0.40
Cl (mEq/L)	99.6 ± 0.7	97.4 ± 0.9
Ca (mEq/L)	10.2 ± 0.1	10.1 ± 0.2
IP (mg/dL)	10.1 ± 0.7	10.1 ± 0.8
T-Cho (mg/dL)	70.4 ± 3.7	87.6 ± 4.5

*sBP* systolic blood pressure; *HR* heart rate; *BW* body weight; *TP* total protein; *Alb* albumin; *BUN* blood urea nitrogen; *Cr* creatinine; *UA* uric acid; *Na* sodium; *K* potassium; *Cl* chloride; *Ca* calcium; *IP* inorganic phosphate; *T-Cho* total cholesterol. Values are mean ± SEM. *P < 0.05 vs DahlS rats fed normal salt by unpaired *t*-test

^a^One-way analysis of variance (ANOVA) followed by Tukey’s honestly significant difference test was used to compare kidney weights. ^†^*P* < 0.05 vs the same side kidney of DahlS rats fed normal salt by Tukey’s honestly significant difference test

### mRNA, protein, and histological analysis

**Fig. 1:**
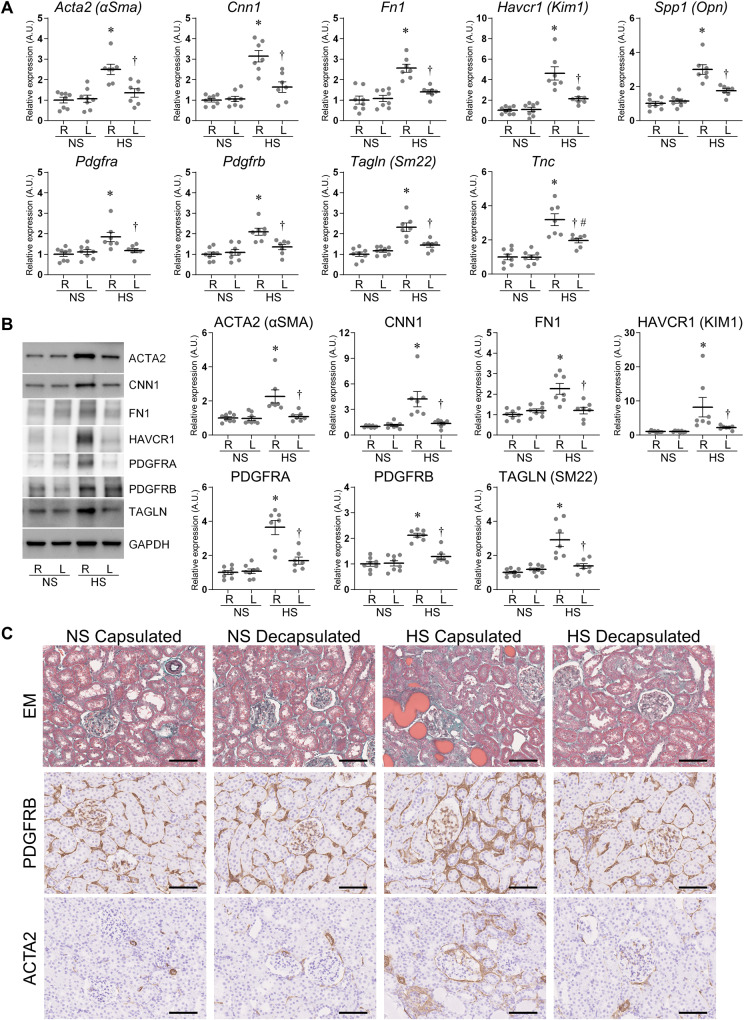
Molecular analysis in the cortex of the normal salt (NS) fed group and high salt (HS) fed group. **A** The mRNA expression levels of *Acta2* (*αSma*), *Cnn1*, *Fn1*, *Havcr1* (*Kim1*), *Spp1* (*Opn*), *Pdgfra*, *Pdgfrb*, *Tagln* (*Sm22*), and *Tnc* were assessed by reverse transcription real-time quantitative polymerase chain reaction in the cortex. The relative mRNA levels were normalized to *Rplp2*, *Ppia*, and *Pgk1* levels. **B** The protein expression of ACTA2, CNN1, FN1, HAVCR1, PDGFRA, PDGFRB, and TAGLN was assessed by western blotting in the cortex. The relative protein levels were normalized to GAPDH level. The data show individual values and mean ± SEM; The value of 1 was assigned to the relative expression of the right contralateral non-decapsulated kidney in the NS group; NS *n* = 8; HS *n* = 7; R, right contralateral non-decapsulated kidney; L, left decapsulated kidney; A.U., arbitrary unit. *P < 0.05 versus the right contralateral non-decapsulated kidney in the NS group; ^†^*P* < 0.05 versus the right contralateral non-decapsulated kidney in each group; ^#^*P* < 0.05 versus the left decapsulated kidney in the NS group by Tukey test. **C** Representative histological images in the cortex stained for Elastica-Masson (EM), PDGFRB, and ACTA2. Scale bar = 100 μm. Capsulated, right contralateral non-decapsulated kidney; Decapsulated, left decapsulated kidney

**Fig. 2:**
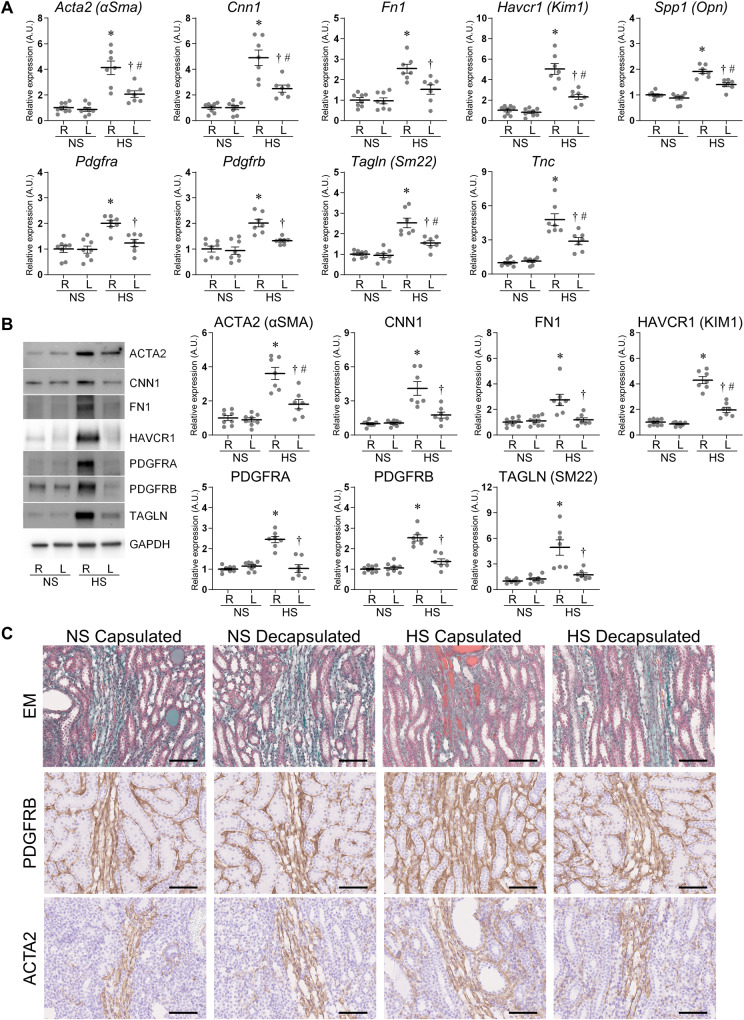
Molecular analysis in the outer medulla of the normal salt (NS) fed group and high salt (HS) fed group. **A** The mRNA expression levels of *Acta2* (*αSma*), *Cnn1*, *Fn1*, *Havcr1* (*Kim1*), *Spp1* (*Opn*), *Pdgfra*, *Pdgfrb*, *Tagln* (*Sm22*), and *Tnc* were assessed by reverse transcription real-time quantitative polymerase chain reaction in the outer medulla. The relative mRNA levels were normalized to *Rplp2*, *Ppia*, and *Pgk1* levels. **B** The protein expression of ACTA2, CNN1, FN1, HAVCR1, PDGFRA, PDGFRB, and TAGLN was assessed by western blotting in the outer medulla. The relative protein levels were normalized to GAPDH level. The data show individual values and mean ± SEM; The value of 1 was assigned to the relative expression of the right contralateral non-decapsulated kidney in the NS group; NS *n* = 8; HS *n* = 7; R, right contralateral non-decapsulated kidney; L, left decapsulated kidney; A.U., arbitrary unit. *P < 0.05 versus the right contralateral non-decapsulated kidney in the NS group; ^†^*P* < 0.05 versus the right contralateral non-decapsulated kidney in each group; ^#^*P* < 0.05 versus the left decapsulated kidney in the NS group by Tukey test. **C** Representative histological images in the outer medulla stained for Elastica-Masson (EM), PDGFRB, and ACTA2. Scale bar = 100 μm. Capsulated, right contralateral non-decapsulated kidney; Decapsulated, left decapsulated kidney

The mRNA expression levels of fibrosis markers *Actin alpha 2* (*Acta2*; also known as *Alpha-smooth muscle actin* (α-*Sma*)), *Calponin 1* (*Cnn1*), *Fibronectin* (*Fn1*), and *Tenascin-C* (*Tnc*); kidney injury markers *Kidney injury molecule 1* (*Kim1*; also known as *Hepatitis A virus cellular receptor 1* (*Havcr1*)) and *Osteopontin* (Opn; also known as *Secreted phosphoprotein 1* (*Spp1*)); and PMT markers *Pdgfra*, *Pdgfrb*, and *Transgelin* (*Tagln*; also known as *Smooth muscle protein 22-alpha* (Sm22)) were significantly increased in the cortex of HS-fed DahlS rats’ right contralateral non-decapsulated kidneys, compared to NS-fed ones (Fig. 1A). The protein expression levels of ACTA2, CNN1, FN1, KIM1, PDGFRA, PDGFRB, and TAGLN were also upregulated in the cortex by HS intake (Fig. 1B). The expression of these markers was significantly attenuated by decapsulation at both the mRNA and protein levels. Similar to the cortex, elevation of fibrosis, kidney injury, and PMT markers by HS intake and benefits of decapsulation were observed in the outer medulla (Fig. 2A, B).

Hyperplasia of the extracellular matrix, renal fibrosis, tubular injury, and PMT markers were detected in the right contralateral non-decapsulated kidneys of DahlS rats fed HS (Fig. 1C and 2C, Supplementary Fig. 2). The interstitial fibrosis and tubular casts were sporadically observed in the right contralateral non-decapsulated kidneys of DahlS rats fed HS by Elastica-Masson staining. The positive area of PDGFRB, ACTA2, and CNN1 was also increased in the right contralateral non-decapsulated kidney of DahlS rats fed HS. Moreover, KIM1 and OPN were stained in the atrophic tubules of the HS groups. Decapsulation ameliorated these markers, similar to the results of the RT-qPCR and western blot. Immunofluorescence staining for Desmin (DES), Nephrin (NPHS1), and Podocin (NPHS2) showed glomerular injury in DahlS rats fed HS, which was improved by decapsulation (Supplementary Fig. 3).

### Morphological changes around the vasa recta

**Fig. 3:**
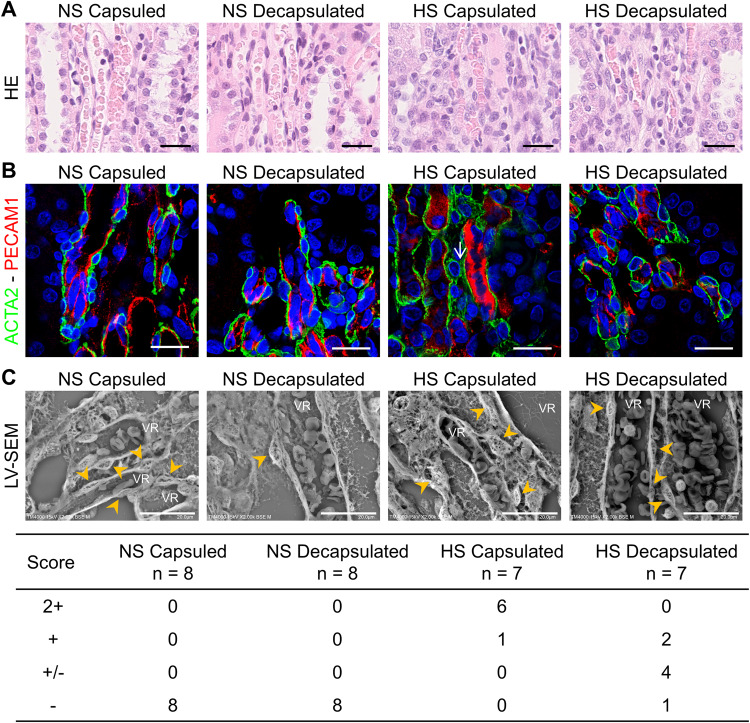
Pericyte structure in the descending vasa recta (VR) of the normal salt (NS)fed group and high salt (HS)fed group. **A** Representative images of hematoxylin-eosin (HE) staining around the vasa recta. Scale bar = 25 µm. **B** Representative immunofluorescence images for ACTA2 (green) and PECAM1 (red) around the vasa recta. Nuclei were stained with Hoechst 33342 (blue). Arrow, ACTA2-positive anomaly layer. Scale bar = 20 μm. **C** Top: Representative images of pericytes (arrowhead) obtained by low-vacuum scanning electron microscopy (LV-SEM). Scale bar = 20 µm. Capsulated, right contralateral non-decapsulated kidney; Decapsulated, left decapsulated kidney. Bottom: Cross-table of pericyte detachment scores in the normal salt-fed group and high salt-fed group. *P* < 0.001 by Fisher’s exact test

Although pericyte detachment was hardly detectable by HE staining, pericyte abnormalities were observed by double-labeling immunofluorescence staining of pericyte marker ACTA2 and vascular endothelial marker PECAM1 (CD31) (Fig. 3A, B). LV-SEM revealed pericyte detachment in the right contralateral non-decapsulated kidneys of DahlS rats fed HS (Fig. 3C). This phenomenon in HS intake was ameliorated even in the expanded vasa recta of the left decapsulated kidneys. Pericyte detachment score was increased in the right contralateral non-decapsulated kidneys of DahlS rats fed HS, and improved by decapsulation (*P* < 0.001 by Fisher’s exact test). DahlS rats fed NS had no pericyte detachment in both the non-decapsulated and decapsulated kidneys. Furthermore, in contrast to DahlS rats, pericyte detachment was not seen in the kidneys of SHR until 14 weeks of age (Supplementary Fig. 4).

**Fig. 4:**
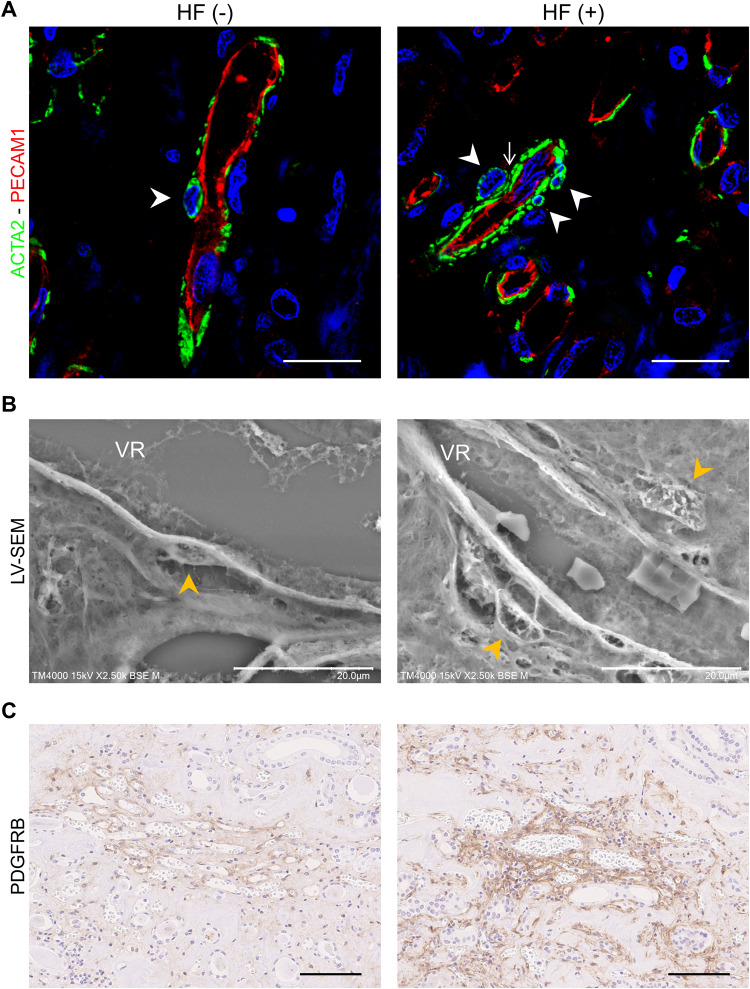
Pericyte structure in human autopsy kidneys from patients with heart failure (HF (+)) and without heart failure (HF (-)). **A** Representative immunofluorescence images for ACTA2 (green) and PECAM1 (red) around the vasa recta. Nuclei were stained with Hoechst 33342 (blue). Arrowhead, pericytes; Arrow, ACTA2 positive anomaly layer. Scale bar = 20 μm. **B** Representative images of pericytes (arrowhead) in the descending vasa recta (VR) were captured by low-vacuum scanning electron microscopy. Scale bar = 20 μm. **C** Representative histological images for PDGFRB around the vasa recta. Scale bar = 100 μm

A total of 8 pathological anatomies were performed between September 2017 and March 2023 in our department (Table 2). Among these, four cases had a history of heart failure. These patients had over 20 mm in IVC diameter, a marker of venous volume expansion [31]. Double staining of ACTA2 and PECAM1 (CD31) identified pericyte abnormalities in patients with heart failure (Fig. 4A). Pericyte detachment was observed in 3/4 of patients with heart failure in LV-SEM, while no pericyte detachment was detected in patients without heart failure (Table 2, Fig. 4B). The PDGFRB-positive area was increased in the interstitial space around the vasa recta in patients with heart failure (Fig. 4C).

**Table 2:** Patient characteristics

No.	Sex	Age	BMI	Cr (mg/dL)	Heart failure	IVC diameter (mm)	Underlying disease	BNP (pg/mL)	EF (%)	Pericyte detachment score
1	M	90	21.2	5.27	+	24.3	HT, DM, CI	3925.1	45.0	2+
2	F	89	23.6	10.9	-	17.7	CRF	206.0	60.5	-
3	M	74	16.8	7.04	-	7.8	DM	154.2	NA	-
4	M	81	21.0	6.83	-	17.2	DM	NA	NA	-
5	F	81	21.7	5.80	+	23.6	DM, CRF	1824.8	58.8	+/-
6	M	92	23.7	3.80	+	28.4	IHD, PMI, AAA	1340.1	26.1	-
7	F	83	18.2	5.35	+	23.3	AAV, ICH	2015.8	37.5	+
8	M	76	18.0	4.85	-	9.2	CCE	132.0	58.9	-

*BMI* body mass index; *Cr* creatinine; *IVC* inferior vena cava; *BNP* brain natriuretic peptide; *EF* ejection fraction; *M* male; *F* female; *HT* hypertension; *DM* diabetes mellitus; *CI* cerebral infarction; *CRF* chronic renal failure; *IHD* ischemic heart disease; *PMI* pacemaker implantation; *AAA* abdominal aortic aneurysm; *AAV* antineutrophil cytoplasmic antibody (ANCA)-associated vasculitis; *ICH* intracerebral hemorrhage; *CCE* cholesterol crystal embolization; *NA* not available

## Discussion

The present study showed pericyte detachment in the kidneys of DahlS rats fed HS, which causes renal congestion accompanying hypertensive heart failure. This was observed in the congested kidneys of our artificial renal congestion model [12, 13]. Renal capsule decapsulation improved pericyte detachment and HS intake-induced renal damage. These results indicated that pericyte detachment was involved in renal congestion. Furthermore, this is the first study to observe pericyte ultrastructure in human kidney samples. Patients with congestive heart failure, which occur in CVP elevation and consequent renal congestion, displayed pericyte detachment in the kidneys. To the best of our knowledge, no reports show ultrastructural or immunohistochemical photographs of pericyte loss and detachment in the human kidneys.

Renal congestion is an important factor in worsening renal function in patients with heart failure. We have recently developed a rat unilateral renal venous congestion model by ligating the vena cava between renal veins [12]. In this model, interstitial fibrosis, higher expression of tubular injury markers, pericyte detachment, and PMT around the vasa recta were found in the left congested kidney. Moreover, reduced RIHP by capsulotomy [12] or suppression of PMT by inhibiting the PDGFR pathway [13] protected against renal interstitial fibrosis induced by renal congestion. Given the above results, our previous artificial renal congestion model would assist in better understanding the unrevealed mechanisms caused by renal venous congestion per se. However, because the right contralateral kidney had a normal renal function and the effects of hemodynamic changes and neuro/hormonal transmitters were limited [12], the results of this model are not directly extended to the aggravation of renal function associated with the progression of heart failure. Therefore, we examined the pericyte structures and molecules linked to PMT in DahlS rats fed HS, which develop venous congestion and show both cardiac and renal failure in the clinical course.

### DahlS rats and renal congestion

DahlS rats with HS diet develop hypertension, congestive heart failure, fluid volume overload, and elevated CVP and RVP, causing renal injury and fibrosis with renal congestion [22–26]. The area of fibrosis was correlated not with the systolic blood pressure but significantly with renal medullary pressure, which is closely related to CVP in DahlS rats [28]. In addition, tolvaptan, a vasopressin receptor 2 antagonist, improved the renal function of rats and humans with heart failure due not to antihypertensive effects but renal decongestion [28, 32]. Sodium-glucose co-transporter 2 inhibitor also reduced CVP, renal medullary perfusion pressure, and cast formation in DahlS rats with high salt loading, whereas loop diuretics only reduced CVP and did not improve renal medullary perfusion pressure and cast formation [33]. The present study showed that renal capsule decapsulation attenuated renal damage in Dahl rats fed HS. Thus, these reports and our results suggest that renal decongestion has a renoprotective potential in hypertensive heart failure.

### Pericyte detachment and renal congestion-induced damage

We found pericyte detachment for the first time in humans. Pericyte detachment was observed only in patients with heart failure. Furthermore, DahlS rats fed HS also had pericyte detachment, but SHR did not show it even at 14 weeks old. SHR, which do not show venous congestion, have a similar magnitude and duration of hypertension as DahlS rats, but show little or slow progression of renal injury [34–37]. Pericyte detachment induces interstitial fibrosis accompanying PMT [19]. Pericytes experiencing PMT exhibited increased myofibroblast markers, including PDGFRs, upregulated collagen deposition, and increased migration away from the vasculature [38–40]. Therefore, renal congestion due to CVP and RVP elevation would induce pericyte detachment rather than arterial hypertension.

Pericytes, covering 10–50% of the vascular surface, are crucial for the stabilization and development of the vascular network [20, 41, 42]. The ratios of pericytes to endothelial cells were 1:1, 1:2.5, and 1:100 in the retina, kidney, and skeletal muscle, respectively [17, 43]. Renal pericytes have multiple functions in renal function and pathology [16–20]. Pericytes maintain proper blood flow and pressure by regulating microvessel diameter through contraction and relaxation [20, 44]. Across the PMT, pericytes become a source of myofibroblasts in renal interstitial fibrogenesis [17–19].

The pathophysiological importance of pericytes has been established in the kidney of several animal models [12, 13, 16–21, 45–47], and in the brain and retina of human diseases [48–50]. Several renal injuries, including unilateral ureteral obstruction, renal artery stenosis, and ischemia-reperfusion injury, induce pericyte detachment [45–47]. Angiotensin II-induced superoxide diffused from the medullary thick ascending limb to pericytes in DahlS rats [51, 52]. Renal NG2-positive pericytes had an M2-macrophage-like ability and contribute to the recovery process after ischemia-reperfusion injury [53]. Single-cell RNA sequencing in humans and mice showed that myofibroblasts predominantly derive from pericytes and fibroblasts [17, 38, 41]. Exogenous administration of renal pericytes protected the kidneys from ischemic damage, suggesting their renoprotective properties [54]. Furthermore, inhibition of the PDGFR pathway suppressed renal interstitial fibrosis in our artificial renal congestion model [13]. Taken together, targeting the pericyte and its detachment could inhibit the progression of renal damage in renal congestion.

### Releasing renal congestion and pericyte protection

Decapsulation protected renal damage in DahlS rats fed HS in the present study. This is consistent with our previous report showing attenuation of PMT-related proteins in the medulla of artificially congested kidneys [12]. Moreover, pericytes were still attached in the decapsulated kidneys of DahlS rats fed HS. The renal capsule is a thin, tough, and fibrous connective tissue and keeps RIHP [55, 56]. We have previously shown that renal perfusion pressure responses to RIHP, natriuresis, and diuresis were attenuated by decapsulation, but medullary blood flow was not affected [57]. In addition, decapsulation ameliorated the increase in RIHP, secretion of hydrogen peroxide into the interstitial space, and production of cortical 20-hydroxyeicosatetraenoic acid, which were induced by increased renal perfusion pressure [58–62]. Thus, these physical and non-physical influences may trigger pericyte detachment and PMT.

Our results and the above evidence may indicate that renal capsule decapsulation is a promising strategy for the protection of the pericytes and eventually against worsening renal function. Decapsulation showed renoprotective effects against ischemic acute kidney injury in piglets [60]. Furthermore, a recent concept called “renal tamponade hypothesis”, in which the renal structure is compressed due to limited space for expansion, has been proposed, and renal capsule decapsulation could be effective for renal congestion-induced injury [63]. However, the kidney capsule presents functional mesenchymal stromal cells in humans and rodents [55, 64]. The immediate recovery of the capsule in humans was known almost 100 years ago, when this surgery was widely practiced [65]. The effect of decapsulation was also transient in this study. A new capsule was found at tissue sampling 7 days after capsulotomy, and kidney weight was not different between the right contralateral non-decapsulated and left decapsulated kidneys. Thus, the suitability of decapsulation in humans would be limited to cases of transient RIHP reduction, such as Page kidney. Other approaches are necessary to protect pericytes for long periods from elevated RIHP in cardiorenal syndrome.

### Limitations

There are several limitations. Firstly, the causal relationship between pericyte detachment and renal impairment has not been established in both rodents and humans. No pericyte detachment in SHR and the mechanism of PMT may imply that pericyte detachment is a cause of renal interstitial injury and fibrosis. However, we cannot deny the possibility that renal injury begins first. Time-course experiments only increase hydrostatic pressure in vivo and in vitro would be needed. Secondly, in the present study, the right non-decapsulated kidney of DahlS rats fed HS had glomerular injury and tubular casts, which means that urinary protein is more abundant in the kidney damaged by renal congestion. Several factors, including changes in renal blood flow, arteriolosclerosis and glomerular injury, as well as pericyte detachment, are related to renal damage caused by renal congestion. Thirdly, the human kidneys examined in this study were donated and the clinical information, such as the value of venous pressure and the information on other renal injuries, was lacking. Pericyte detachment was reported in the kidneys of several experimental animal models [45–47]. No clinical markers have been identified in urine and plasma that reflect only pericyte detachment itself. Elevated RIHP, which is a trigger of pericyte detachment and PMT, also induces tubular injury and the secretion of oxidative stress and chemical mediators from tubular cells into the interstitial space and urine in experimental animals [47, 57, 61]. Urinary markers such as oxidative stress and KIM1 may be indicators of pericyte detachment and PMT. Future studies are necessary to examine living human kidneys in detail to dissect the pathophysiological roles and clinical markers of pericyte detachment.

## Conclusion

Pericyte detachment is involved in the renal tubulointerstitial injury and fibrosis of rats and humans with renal congestion. Although arterial hypertension has been traditionally emphasized in the pathophysiology of renal damage, the venous congestion accompanying venous hypertension must also be focused on. Further understanding of this concept may lead to novel therapeutic strategies for worsening renal function.

### Supplementary information

Supplementary information
