# Supplementary material for: Pericyte detachment and renal congestion involve interstitial injury and fibrosis in Dahl salt-sensitive rats and humans with heart failure
Source: Hypertens Res. 2023 Oct 16;46(12):2705–17. doi: 10.1038/s41440-023-01451-3 (PMC10695822; doi:10.1038/s41440-023-01451-3)
Supplement: Supplementary file 1 — Supplementary information [file 41440_2023_1451_MOESM1_ESM.pdf]

Supplementary information

Pericyte detachment and renal congestion involve interstitial injury and fibrosis in Dahl salt-sensitive rats and humans with heart failure

Hiroki Ito[1,2], Takuo Hirose[2,3,*], Shigemitsu Sato[3], Chika Takahashi[3], Risa Ishikawa[1], Akari Endo[1,2], Ayaka Kamada[1], Ikuko Oba-Yabana[1], Tomoyoshi Kimura[1], Kazuhiro Murakami[4], Yasuhiro Nakamura[4], Kazuhiro Takahashi[2], Takefumi Mori[1,3,*]

1 Division of Nephrology and Endocrinology, Faculty of Medicine, Tohoku Medical and Pharmaceutical University, Sendai, Japan
2 Department of Endocrinology and Applied Medical Science, Tohoku University Graduate School of Medicine, Sendai, Japan
3 Division of Integrative Renal Replacement Therapy, Faculty of Medicine, Tohoku Medical and Pharmaceutical University, Sendai, Japan
4 Division of Pathology, Tohoku Medical and Pharmaceutical University Hospital, Sendai, Japan

Short title: renal congestion and pericyte detachment

*Corresponding author
Takuo Hirose, Ph.D.
Department of Endocrinology and Applied Medical Science
Tohoku University Graduate School of Medicine
2-1, Seiryo, Aoba, 980-8575 Sendai, Japan
Tel/Fax: +81-22-717-7482
E-mail: hirose-t@med.tohoku.ac.jp
ORCID: 0000-0002-8761-3779

Takefumi Mori, M.D., Ph.D.
Division of Nephrology and Endocrinology, Faculty of Medicine,
Tohoku Medical and Pharmaceutical University
1-15-1, Fukumuro, Miyagino, 983-8536, Sendai, Japan
Tel: +81–22–259–1221
Fax: +81–22–259–1232
E-mail: tmori@tohoku-mpu.ac.jp

Supplementary Figure 1

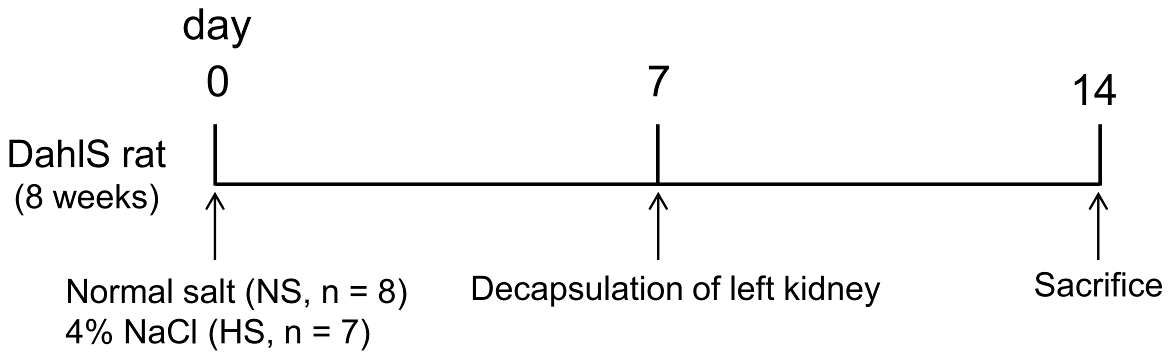

Supplementary Figure 1. Experimental design. Male Dahl salt-sensitive rats (DahlS; SS/Jr/Mcwi) were divided into two groups: normal salt (NS; 0.4% NaCl diet, n = 8) and high salt (HS; 4% NaCl diet, n = 7) (Day 0). Decapsulation, which completely removed the renal capsule to reduce renal interstitial hydrostatic pressure, was performed only on the left kidney (Day 7). Tissues were collected after blood sampling and the rats were euthanized under deep anesthesia (Day 14). No rats died during the experimental period.

Supplementary Figure 2

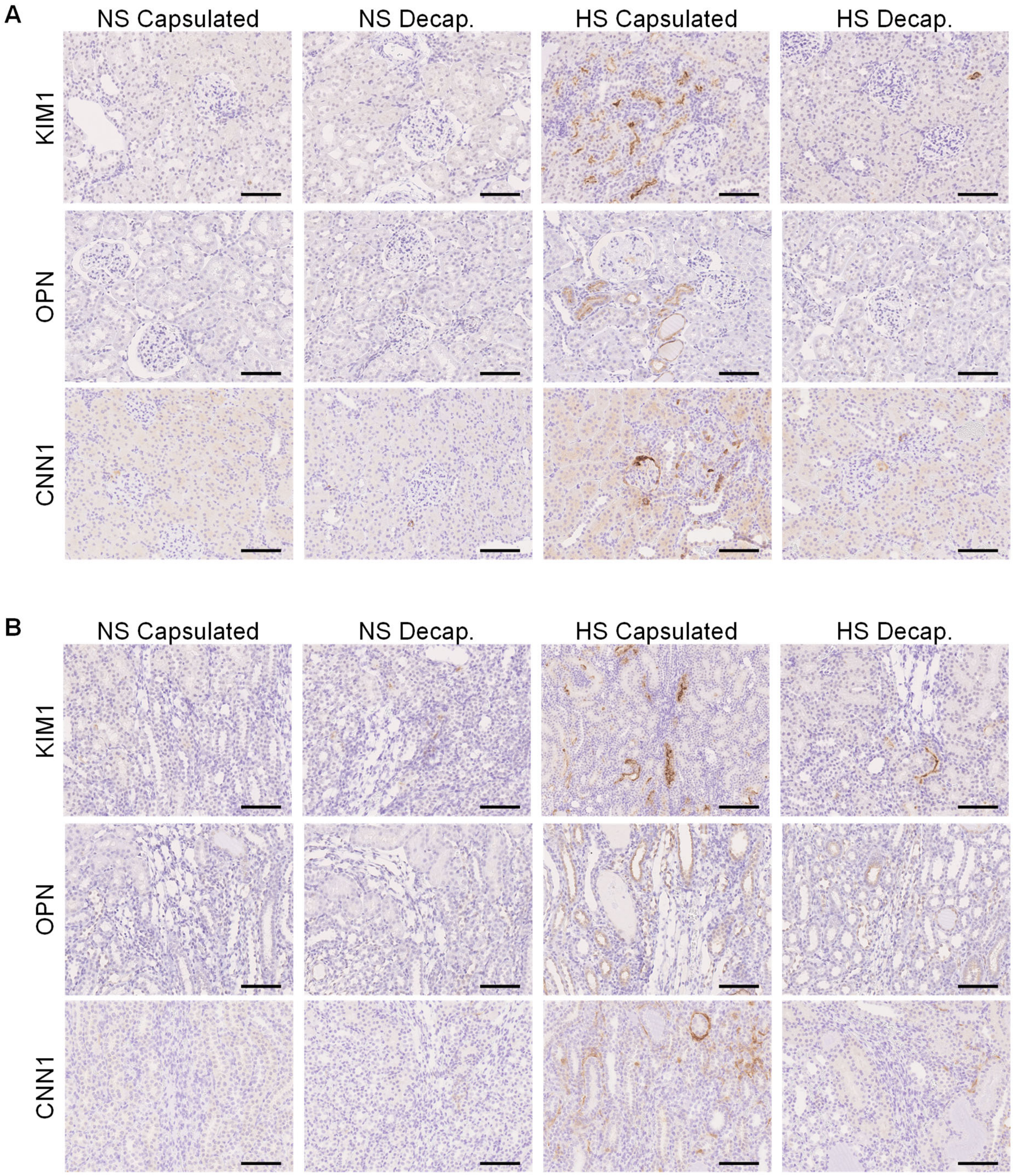

Supplementary Figure 2. Immunostaining of KIM1, OPN, and CNN1 in the cortex (**A**) and the outer medulla (**B**) of the normal salt (NS) and high salt (HS) fed groups. Scale bar = 100 μm. Capsulated, right contralateral non-decapsulated kidney; Decap., left decapsulated kidney.

Supplementary Figure 3

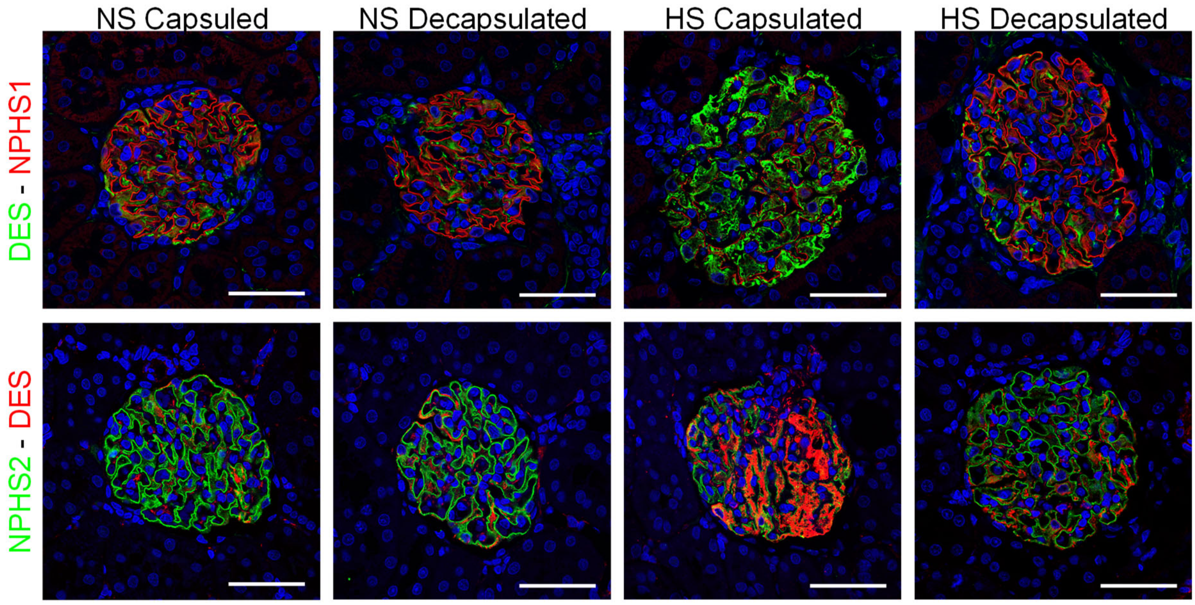

Supplementary Figure 3. Immunofluorescence staining of DES, NPHS1 (Nephrin), and NPHS2 (Podocin) in the glomerulus of the normal salt (NS) and high salt (HS) fed groups. Nuclei were stained with Hoechst 33342 (blue). Scale bar = 50 µm. Capsulated, right contralateral non-decapsulated kidney; Decap., left decapsulated kidney.

Supplementary Figure 4

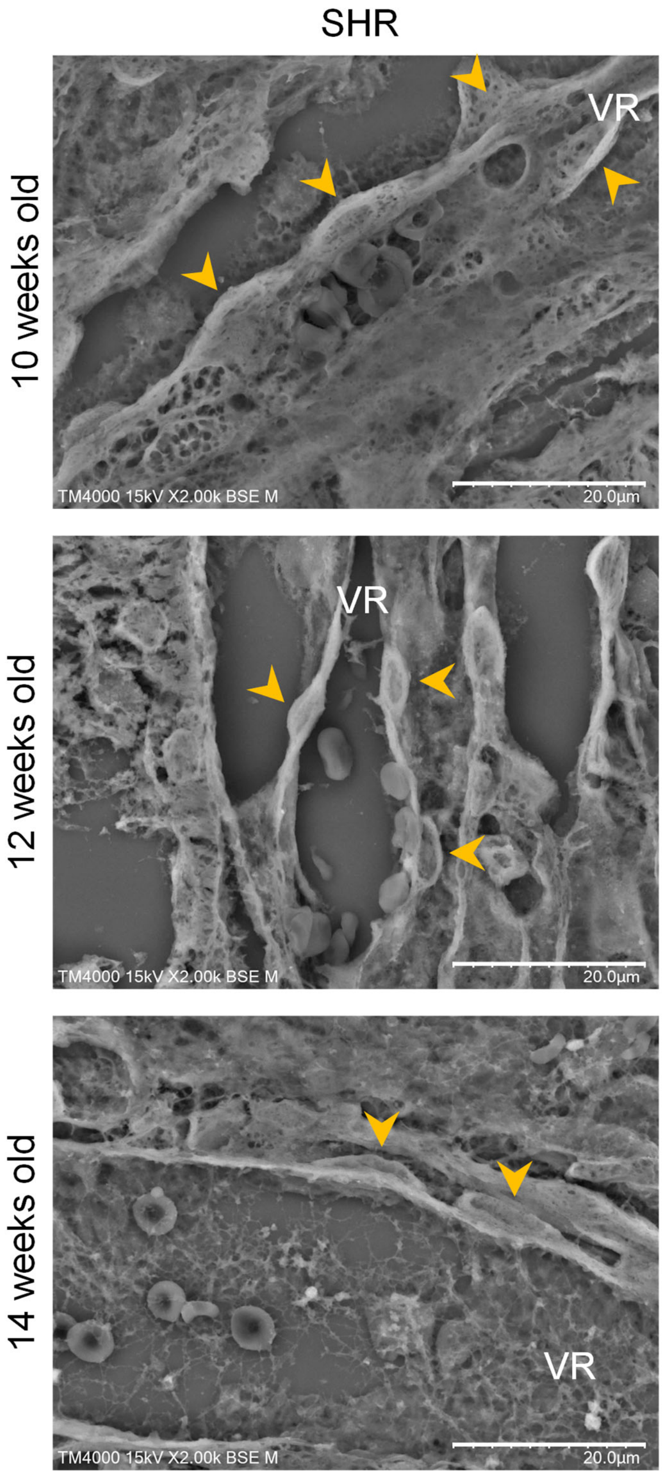

Supplementary Figure 4. Pericyte structure (arrowhead) in the descending vasa recta (VR) of spontaneously hypertensive rats (SHR) by low-vacuum scanning electron microscopy. Scale bar = 20 μm.

Supplementary Table 1: Primer information.

Symbol	primer set ID* or Forward primer / Reverse primer (5’-3’)**
Acta2 (*αSMA*)	RA060203
Cnn1	CCTGTCTGCTGAGGTAAAGAACA / CCGGCTGGAGCTTGTTGATAAAT
Fn1	RA055827
Havcr1 (*Kim1*)	RA057664
Pdgfra	RA059727
Pdgfrb	RA048695
Pgk1	ATGGAGCCAAGTCGGTTGTG / GGTTGGCACAGGCATTCTCT
Ppia	CCACCGTGTTCTTCGACATC / CGTGTGAAGTCACCACCCTG
Rplp2	RA015377
Spp1 (*Opn*)	RA017345
Tagln (*Sm22*)	RA063024
Tnc	AACGAACTGCCCACATCTCG / TTCCGGTTCAGCTTCTGTGG

*RA XXs was purchased from Takara Bio.

**Primers were designed by Primer3 (http://primer3.ut.ee) and synthesized by Integrated DNA Technologies.

Supplementary Table 2: Antibody information.

Antigen	Company	Catalog #	Clone	Host	WB	IHC	IF
ACTA2 (αSMA)	Cell Signaling	19245S	D4K9N	rabbit	1:1000	1:500**	1:200**
CNN1	Sigma-Aldrich	C2687	hCP	mouse	1:1000	1:500*	
DES	Abcam	ab15200		rabbit			1:400**
DES	Santa Cruz	sc-7579		mouse			1:200*
FN1	Merck	F3648		rabbit	1:1000		
GAPDH	Cell Signaling	2118S	14C10	rabbit	1:5000		
HAVCR1 (KIM1)	R&D system	AF3689		goat	1:1000	1:1250*	
NPHS1 (Nephrin)	PROGEN	GP-N2		guinea pig			1:500**
NPHS2 (Podocin)	IBL	29040		rabbit			1:100*
NG2	Millipore	AB5320		rabbit			1:500**
PECAM1 (CD31)	Santa Cruz	sc-1506		goat			1:50**
PDGFRA	Cell Signaling	3174S	D1E1E	rabbit	1:1000		
PDGFRB	Abcam	ab32570	Y92	rabbit	1:1000	1:200**	
SPP1 (OPN)	Santa Cruz	sc-10591		goat		1:100*	
TAGLN (SM22)	Abcam	ab14106		rabbit	1:1000		

*The antigens were retrieved by autoclave heating for 5 min in 10 mmol/L citrate buffer (pH 6.0).

**The antigens were retrieved by autoclave heating for 5 min in 1.0 mmol/L ethylenediaminetetraacetic acid buffer (pH 9.0).
